# Test-retest variability of high resolution positron emission tomography (PET) imaging of cortical serotonin (5HT_2A_) receptors in older, healthy adults

**DOI:** 10.1186/1471-2342-9-12

**Published:** 2009-07-06

**Authors:** Tiffany W Chow, David C Mamo, Hiroyuki Uchida, Ariel Graff-Guerrero, Sylvain Houle, Gwenn S Smith, Bruce G Pollock, Benoit H Mulsant

**Affiliations:** 1 The Rotman Research Institute of Baycrest Centre for Geriatric Care, Toronto, ON, Canada; 2 Division of Neurology, Department of Medicine, University of Toronto, Toronto, ON, Canada; 3 Department of Psychiatry, University of Toronto, Toronto, ON, Canada; 4 Centre for Addiction and Mental Health, Toronto, ON, Canada

## Abstract

**Background:**

Position emission tomography (PET) imaging using [^18^F]-setoperone to quantify cortical 5-HT_2A _receptors has the potential to inform pharmacological treatments for geriatric depression and dementia. Prior reports indicate a significant normal aging effect on serotonin 5HT_2A _receptor (5HT_2A_R) binding potential. The purpose of this study was to assess the test-retest variability of [^18^F]-setoperone PET with a high resolution scanner (HRRT) for measuring 5HT_2A_R availability in subjects greater than 60 years old. Methods: Six healthy subjects (age range = 65–78 years) completed two [^18^F]-setoperone PET scans on two separate occasions 5–16 weeks apart.

**Results:**

The average difference in the binding potential (BP_ND_) as measured on the two occasions in the frontal and temporal cortical regions ranged between 2 and 12%, with the lowest intraclass correlation coefficient in anterior cingulate regions.

**Conclusion:**

We conclude that the test-retest variability of [^18^F]-setoperone PET in elderly subjects is comparable to that of [^18^F]-setoperone and other 5HT_2A_R radiotracers in younger subject samples.

## Background

As the proportion of the aged in the population increases, cognitive impairment and depression in older adults has become a public health priority. Twenty-five to thirty percent of nursing home residents are taking second generation antipsychotic medications [1], for which the serotonin 2A receptor (5HT_2A_R) is a target of action. In addition, selective serotonin reuptake inhibitors (SSRIs) are first line drugs in the management of depression and have recently been studied for the management of psychological and behavioural manifestations of dementia [2,3]. The efficacy and/or adverse effects of atypical antipsychotics (e.g., risperidone [4]; and SSRIs in these contexts is predicated upon pre- and post-synaptic effects at several central nervous system serotonin receptors, so that the ability to quantify serotonin receptors in vivo in older subjects is critical to understanding more about the mechanisms of action of the available medications and to inform the development of more effective treatments.

Autoradiography findings [5-7] and radiotracer PET studies [8-15] have reported both increases and decreases in 5HT_2A_R in major depression in younger patients, but no change in older depressed patients (in one study), and decreases in Alzheimer's disease (AD). In addition to the reported 5HT_2A_R reduction due to neuropsychiatric disorders, there are significant declines in 5HT_2A_R binding in normal aging, that are independent of disease state [8,12]. Given the decrease in 5HT_2A_R binding with age and disease, it is important to assess the stability of 5HT_2A_R measurements prior to undertaking studies to measure disease and treatment related effects for older subjects.

The radiotracers [^18^F]-altanserin, [^11^C]-MDL100,907, and [^18^F]-setoperone have been used to study cortical 5HT_2A_R in neurochemical PET imaging studies [12,14,16-21]. Test-retest variability of 5HT_2A_R measurement with [^18^F]-altanserin and PET with arterial blood sampling has been reported in several studies. Soares et al. reported high mean intra-subject % change of 11–14% for cortical brain areas for the ratio of specific to non-displaceable brain uptakes and the ratio of specific brain uptake to total parent plasma concentration [21]. A four compartment mode tracer kinetic model yielded average differences of 13% or less in regions of high receptor concentrations and 16–20% for regions of low receptor concentrations. The Logan graphical analysis method showed variability of 12% or less across ratios for distribution volumes and of less than 10% for distribution volume ratios normalized to the cerebellum [22]. The observation that radiolabelled metabolites of [^18^F]-altanserin cross the blood brain barrier complicates the use of the cerebellum as the input function as opposed to arterial blood [23,24]. We used setoperone to offer our subjects a less invasive procedure. Kapur et al. [19] found even lower mean intra-subject percent change and higher intra-class correlation coefficients for [^18^F]-setoperone using the cerebellum as the input function. This degree of reliability has been shown thus far only in young subjects (under age 40 years) for [^18^F]-setoperone [19]. We followed up on this previous work by testing the reliability of measuring 5HT_2A_R in healthy elderly subjects (over age 60 years) using the radioligand [^18^F]-setoperone with a high resolution brain PET scanner (HRRT). We chose to examine this reliability in areas of the brain related to depression and dementia: prefrontal, including the anterior cingulate gyrus, temporal, and insular cortex.

## Methods

Healthy subjects volunteered in response to advertisements in the community. The inclusion criteria included: minimum age of 60 years; independence in all activities of daily living; and absence of any current or past psychiatric illness, significant neurological disorder (e.g., stroke) or diagnosis of cognitive impairment. Subjects were excluded if they had a history of substance abuse or dependence, unstable systemic disease, concurrent use of psychotropic drugs including SSRIs, serotonin/norepinephrine reuptake inhibitor antidepressants, trazodone, second generation antipsychotics, or contraindication to MRI procedures. The subjects provided written informed consent as approved by the Research Ethics Board at the Centre for Addiction and Mental Health (CAMH).

Each of 6 subjects completed two [^18^F]-setoperone PET scans separated in time by 5 to 16 weeks (specifically, 5, 5, 6, 11, 13, and 16 week intervals), in addition to a single MRI scan of the brain for the purpose of co-registration and exclusion of brain pathology.

MRI scanning procedure: Participants underwent standard fast spin echo T1-weighted imaging (GE Magnetom (Milwaukee, WI) 1.5 T scanner; fast spoiled gradient echo, time to echo = 5.3–15 msec, repetition time = 8.9–12 msec, field of view = 20 cm (three-dimensional), 256 × 256 voxel 1.5 isotropic, number of excitations = 1) and a proton density image (time to echo = 17, repetition time = 6,000, field of view = 22 cm (two-dimensional), 256 × 256, slice thickness = 2 mm, number of excitations = 2). The T1 image was used for the region of interest (ROI) analysis.

PET scanning procedure: The PET scanning methods have been described previously by Mamo et al. [25] Briefly, subjects fasted from 8 AM on the day of the PET scan until after the study. Upon arrival at the PET Centre, the PET procedures were reviewed with the subject. Patients were scanned lying supine and with fixation of the head achieved using a thermoplastic facemask (Raycast Efficast, Orfit Industries, http://www.orfit.com), allowing for consistent repositioning between the two PET scans. A catheter was then inserted into an antecubital vein for radiotracer administration. Then, transmission scans were acquired immediately before emission scans using a single photon ^137^Cs source for attenuation correction of the emission scans.

[^18^F]-setoperone was prepared by the previous published method of Maziere et al, with [^18^F]-fluoride displacement on the nitro-derivative precursor of setoperone [26]. Dynamic PET scans began immediately upon bolus injection of [^18^F]-setoperone (mean dose = 4.8 mCi [SD = 0.2], mean specific activity = 115.0 mCi/μmol [SD = 975]) and lasted for 90 minutes. A HRRT high-resolution neuro-PET camera system (Siemens Molecular Imaging, Knoxville, TN) was used to acquire the PET data. Emission data were acquired in list mode and later reconstructed by filtered-back projection to yield dynamic images in 22 frames (five 1-minute and seventeen 5-minute frames). The data were reconstructed into 207 brain sections with an interslice distance of 1.2 mm and an in-plane resolution of approximately 2.8 mm full width at half-maximum.

### Image Analysis

The PET data were analysed using a region of interest method described previously (Region of Mental Interest [ROMI]) by Rusjan et al. [27]. This software, in concert with SPM2, (i) transforms a standard brain template with a set of predefined ROIs to match individual high-resolution MR images, (ii) refines the ROIs from the transformed template based on the gray matter probability of voxels in the individual MR images (segmentation step), and (iii) co-registers the individual MR images to the PET images so that the individual refined ROIs are transformed to the PET image space. ROIs delineated automatically with ROMI were left and right: frontal, temporal, anterior cingulate, and insula. The ROI template is based on the anatomical label atlas of Talairach transformed to the standard ICBM//MNI 152 brain, which is included in the WFU toolbox for SPM [28]. One trained investigator (HU) visually checked ROI placement for all subjects and judged all but one of the ROIs delineated automatically to be accurate. The cerebellar ROI used to generate BP_ND_s for the one subject was drawn manually by HU, using the same anatomical landmarks as ROMI.

ROMI was developed after the prior study of setoperone PET test-retest variability [19]. It affords analysis of insular and anterior cingulate regions, which have been of interest in behavioural neuroscience. e.g., [29,30]

Based on previous reports that the cerebellar binding is not significantly displaced by 5HT_2A _antagonists in non-human primates and human subjects [31,32], the cerebellar cortex, excluding the vermis, was used as the reference region. PMOD version 2.7 (PMOD Technologies, Ltd., Zurich, Switzerland) was used to generate time activity curves (TAC). To determine the 5HT_2 _receptor binding potential, we used the simplified reference tissue model (SRTM). Binding potential (BP_ND_) is defined as the ratio of k_3 _to k_4_, where k_3 _and k_4 _are the rate constants of radioligand delivery and transfer out of the specifically bound compartment in a two-tissue compartment model [33].

### Statistical Analysis

We assessed the test-retest reliability of the binding potentials for each of the six ROIs automatically delineated by ROMI (N = 6) using single measure intraclass correlation coefficients (ICC, SPSS: Analysis: Scale: Reliability: Statistics – ICC, two-way mixed effects model for consistency). The interval between scans was factored into the Repeated Measures analysis for ICC as a between-subjects factor. An interval of 8 weeks or fewer was considered short; intervals of 9 weeks or more (maximum 16 for this study) were considered longer. We also examined repeatability coefficients (RC).

The RC was calculated as twice the standard deviation (SD) of the difference between the two BP_ND _values for each of the ROIs from the 1^st ^and 2^nd ^analyses (e.g., left frontal BPs from scans 1A and 1B) [34] Analysis of variance was performed to compare the volume of the ROIs between the first and the second scan.

## Results

Table 1 lists the mean 5HT_2A_R BP_ND_s and volumes for each of the ROIs. Table 2 lists the percent change in mean difference, ICC, and RC for each of the regional 5HT_2A_R BP_ND_s.

**Table 1:** Mean binding potentials (BP_ND_) and standard deviations (*SD*) for repeated setoperone PET scans (N = 6).

Left-sided ROIs	Mean BP_ND _*(SD)*	*Mean volume of ROI *(SD) in mm*^3^	Right-sided ROIs	Mean BP_ND _*(SD)*	
	0.52	8070		0.47	*8641*
Scan 1 frontal	*(0.28)*	(987)	Scan 1 frontal	*(0.25)*	*(999)*

	0.53	8074		0.49	*8661*
Scan 2 frontal	*(0.27)*	(994)	Scan 2 frontal	*(0.24)*	*(1005)*

	0.60	8133		0.58	*8221*
Scan 1 temporal	*(0.37)*	(1082)	Scan 1 temporal	*(0.39)*	*(755)*

	0.58	8115		0.59	*8231*
Scan 2 temporal	(*0.33)*	(1057)	Scan 2 temporal	*(0.34)*	*(774)*

	0.33	741		0.22	*762*
Scan 1 anterior cingulate	(*0.34)*	(138)	Scan 1 anterior cingulate	(*0.25)*	*(135)*

	0.36	735		0.25	*753*
Scan 2 anterior cingulate	*(0.24)*	(140)	Scan 2 anterior cingulate	(*0.20)*	*(123)*

	0.45	*3649*			14221
**Scan 1 average insula	*(0.31)*	*(794)*	Scan 1 cerebellum	Reference ROI	(1821)

	0.48	*3628*			14188
**Scan 2 average insula	*(0.29)*	*(783)*	Scan 2 cerebellum	Reference ROI	(1799)

* Comparison between ROIs' volume (scan 1 vs. scan 2): F[3,8] = 0.108, p = 0.99.

**ROMI generates the insular region of interest as an average of the left and right sides.

**Table 2:** Mean differences, intraclass correlation coefficients (ICC), and repeatability coefficients (RC) for [^18^F]-setoperone binding potential test-retest.

	Left			Right			Average	
**Region of Interest**	**Mean diff**	**ICC [CI]**	**RC**	**Mean diff**	**ICC [CI]**	**RC**	**ICC [CI]**	**RC**

**Temporal lobe**	3.3%	0.97	0.05	1.7%	0.94	0.02	0.96	0.01
		[.83, 1.0]			[.66, .99]		[.72, .99]	

**Frontal lobe**	1.9%	0.94	0.03	4.1%	0.91	0.05	0.93	0.04
		[.69, .99]			[.53, .99]		[.43, .96]	

**Anterior cingulate**	8.3%	0.69	0.06	12.0%	0.71	0.06	.76	0.06
		[-.09, .95]			[-.05, .96]		[.01, .97]	

**Insula**							0.97	
	-	-	-	-	-	-	[.85, 1.0]	0.06

**Average**		0.87	0.05		0.85	0.04	0.91	0.04

All p values for ICC < .05.

Prefrontal, temporal, and insular cortical ROIs had high test-retest reliability (ICCs ranging from .91 to .97). The anterior cingulate ROIs generate low signal, approximately half that of the temporal ROIs, but this may be due to the smaller volumes of those ROIs. Nevertheless, the right anterior cingulate ROIs generate 2/3 the BP_ND _of the left, despite the right side having a larger volume. Test-retest reliability may suffer in ROIs of smaller volume and/or those with lowest radiotracer binding.

Both left and right anterior cingulate ROIs were less consistent between scans, especially for two subjects who showed an anterior cingulate BP_ND _value of zero or unmeasurable from one but not their second PET scans. These two subjects consisted of one 76 year old woman and one 63 year old man, with between-PET intervals of 5 and 6 weeks, respectively. When data from the two subjects were excluded from the analysis of anterior cingulate measures, the new % change in left and right mean difference (2.1% and 5.5%, respectively), RC (.03 and .05, respectively) were lowered to the range of other ROIs for the group. The slight improvement in the right anterior cingulate ICC of .71 to .78 was not statistically significant (p > 0.05).

All subjects in this study were right-handed. This may account for the asymmetry of BP_ND _in anterior cingulate ROIs to some degree.

The deviation from one scan to the second was not significantly related to the short vs. longer time interval between scans (F_[df = 1,5] _= 118.92, p = .07).

## Discussion

The BP_ND_s for frontal and temporal ROIs in this report is comparable to previously published figures: Meltzer et al. found uncorrected BP_ND_s of 0.53–0.59, and Blin et al. reported that most BP_ND_s were near 0.43. These figures fall within our range of 0.33 – 0.6, but the right anterior cingulate BP_ND _in this study seemed to dip close to measures of BP_ND_s from anterior cingulate ROIs in Blin's study on subjects with Alzheimer's disease. Although we excluded subjects who were cognitively impaired, it is possible that the two subjects with the lowest anterior cingulate BP_ND_s are in a pre-symptomatic phase of Alzheimer's disease.

Another consideration for these results is the relative impact of scan resolution on ROIs of differing sizes and shapes [35,36]. Kessler et al. suggest that a ROI with diameter of at least 2.7 × FWHM is the smallest volume to obtain the full radioactivity recovery with inter-slice distance of no more than 1/2 × FWHM on the z axis. We used an HRRT scanner with FWHM of 2.8 mm and inter-slice distance of 1.2 mm. This FWHM is ~4 times lower than the diameter of the anterior cingulate, which was the smallest ROI studied, volume = 740 mm^3 ^(assuming a sphere with diameter 11.2 mm). Thus, our scanner resolution should be able to handle all of the ROIs included in this study.

This study shows that cortical 5HT_2A_R as measured with [^18^F]-setoperone PET and using non-invasive modelling methodology is reproducible to the same extent observed in other test-rest studies of younger subjects. The level of reliability paralleled the prior study by Kapur et al. showing high ICCs (.96–.97) for frontal and temporal regions in subjects aged 21–35 years [19]. An age-related decrease in cortical 5HT2AR (including the anterior cingulate) has been observed on other PET studies of the 5HT_2A_R [8,37]. The low signal in the anterior cingulate in the present study suggests that partial volume correction may be necessary, particularly for between-group comparisons.

The cortical BP_ND_s observed in older subjects were lower than BP_ND_s among younger controls reported previously in the literature by studies that did not complete test-retest scans [8,11,14,37-39]. Our frontal BP_ND_s were approximately 25–27% of the average frontal and temporal BPs reported by Kapur et al. in subjects aged 21–35 and 20% of those reported for the subset of 20–25 year old subjects in the study by Lewis et al. using [^18^F]-setoperone and the same modelling with SRTM but imaging with the lower resolution PET scanner (0.5 vs. 2.5 mm) [20]. Extrapolation into the aged population using the 6%-per-decade decline in altanserin binding reported by Adams et al. [40] could result in BP at roughly 20–25% that of younger subjects, but the average 1.5%-per-decade decline in setoperone binding reported by Blin et al. [8] would extrapolate to a less drastic decline than the low BP_ND_s reported in the current study. Nevertheless, our BP_ND_s are not likely to have resulted merely from a quantification approach that differed from Kapur et al. [19].

To our knowledge, this is the first study to report test-retest variability of 5HT_2A_R BPs in health control subjects over age 60. This is also one of the few reported test-retest studies conducted in an HRRT scanner. The small sample size used in this study may be a limitation and should be considered in interpreting the results. Power analyses for standard beta level = 0.2 were conducted using required sample size , where SD = standard deviation and d = mean difference of BPs from the right temporal ROI, which had the highest ICC in this study. Whereas within-subject differences of 10% or more could be detected with a sample size of 8, between-group differences of 25% or more would require a sample size of 28 subjects in each comparison group. This is a significant increase from the sample size of 12–15 per group extrapolated in Kapur et al.'s previous study in healthy young controls [19]. The sample size in studies seeking between-group differences in 5HT_2A_R BP was likely driven up by the large between-subjects standard deviation among healthy elderly.

## Conclusion

There is high test-retest reliability for healthy elderly subjects using setoperone PET imaging, yet clinical researchers must take high intersubject variability for this population into account when designing studies of the role of 5HT_2A_R as a biomarker for exploring serotonergic function in elderly patients with depression or dementia.

## Competing interests

The authors declare that they have no competing interests.

## Authors' contributions

TWC, DM, HU, AG and GSS participated in the design of the study. TWC, AG, and HU collected the PET imaging data and coordinated the study. SH, BGP, and BHM made substantial contributions to the acquisition of data. AG and HU completed imaging analysis. TWC and HU performed statistical analyses for the test-retest comparisons. TWC and HU drafted and revised the manuscript. All authors read and approved the final manuscript.

## Pre-publication history

The pre-publication history for this paper can be accessed here:

http://www.biomedcentral.com/1471-2342/9/12/prepub
